# Lung cancer and COPD rates in Apulia: a multilevel multimember model for smoothing disease mapping

**DOI:** 10.1186/1476-072X-9-15

**Published:** 2010-03-05

**Authors:** Nicola Bartolomeo, Paolo Trerotoli, Gabriella Serio

**Affiliations:** 1 Department of Biomedical Science and Human Oncology, Chair of Medical Statistics, University of Bari. Bari. Italy

## Abstract

**Background:**

If spatial representations of hospitalization rates are used, a problem of instability arises when they are calculated on small areas, owing to the small number of expected and observed cases. Aim of this study is to assess the effect of smoothing, based on the assumption that hospitalization rates, when calculated at the municipal level, may be influenced by both the neighboring municipalities and the health service organization, as well as by environmental risk factors associated with the disease under study.

**Methods:**

To smooth rates we hypothesize that each municipality belongs to two independent hierarchical levels; at one of these levels subjects may belong to a plurality of superior hierarchical objects. Two different models, so-called Multilevel Multimembership Models, are fitted. In the first the structure of random effects was: the municipality heterogeneity, the spatial dependence of the municipalities and the local health service organization. In the second we replaced the local health service organization effect with the environmental risk effect for each municipality area.

The models were applied to spatially represent the rates of hospitalization for lung cancer and chronic obstructive pulmonary disease, determined through the hospital discharge forms recorded in Apulia for the year 2006.

**Results:**

The effect of smoothing was greater in smaller municipalities and in those with a more unstable Risk Adjusted Rate (RAR) due to the small number of cases and of population at risk. When a hierarchical level representing the ASL is inserted, the model fits the data better.

**Conclusion:**

Maps of hospitalization rates for lung cancer and chronic obstructive pulmonary disease, shaded with the rates obtained at the end of the smoothing procedure, change the visual picture of the disease distribution over the whole territory, and if detected by the model, seem to express a geographical distribution pattern in specific areas of the region. In the case of lung cancer, the models show a clear difference between RAR and smoothed RAR. The inclusion of a random effect indicating the ASL contributed to improve the graphic representation of the results, whereas the environmental risk was not found to be a better hierarchical level than the municipality for fitting of the model.

## Background

Spatial analysis of disease and health care aspects by constructing maps is a useful tool for assessing indicators of disease distribution levels. Geographic analysis makes it possible to analyze what is happening in an entire region, so as to identify the main characteristics of the spatial structure of the epidemiological phenomenon under study. When analyzing a map, it is necessary to find out whether the cases represented are randomly distributed or else the result of a process caused by factors present in the space being studied [1,2].

Spatial analysis is often used to assess mortality or hospitalization rates but in such cases a problem of instability arises when they are calculated on small areas, owing to the small number of expected and observed cases [3]. It is therefore necessary to perform spatial smoothing to prevent spatial analysis from generating an incorrect interpretation of geographic variations of the risks of hospitalization or mortality [4].

While the estimate of risk in any single area is optimal when the location is not seen as relevant and independence across space is assumed, it is possible to derive improved estimates of the relative risk by building estimators that take into account spatial dependence. Spatially smoothed estimates are, therefore, more appropriate for the assessment of geographic variation than those which do not envisage spatial dependence [5].

In the spatial representation of a disease outbreak or epidemiological phenomenon, apart from strictly geographical aspects that can influence the impact, not only can environmental risk factors that are often associated with the disease to be represented have an effect, but also the health service organization, in terms of the territorial management. This is particularly true if the epidemiological analysis is conducted using data sources of a prevalently administrative nature, such as hospital discharge sheets.

The primary aim of this study was to show, by spatial representation, how the hospitalization rate can be influenced both by the immediately neighboring municipalities and by the local health service management (ASL) to which the municipality belongs as well as by environmental risk factors associated with the disease under study.

For this analysis, a *spatial multiple membership model *was used. The Multiple Membership Model is a hierarchical model in which lower level units can be simultaneously members of more than one higher level unit [6].

As examples, the hospitalization rates for lung cancer and for chronic obstructive pulmonary disease (COPD) recorded for the Apulia region were used. In these areas, a high environmental risk and high prevalence, incidence and hospitalization rates are present [7] and need to be correctly identified. The data source used was the Hospital Discharge Forms (HDF) for the year 2006 [8].

## Methods

### Statistical analysis

To estimate the spatial effects with a multilevel model, the model must contain two components specifying the structure of random effects: a random effect or heterogeneity term, and a term representing the spatial contribution of neighborhood areas (clustering).

Let's consider the i-th municipality with Oi observed cases and Ei expected cases obtained at the end of a Risk Adjustment procedure by gender, as well as age grouped into eight classes (0, 1-4, 5-14, 15-24, 25-44, 45-64, 65-74, >74). For the Risk Adjustment we used a logistic model, and the fitting measure was the c-statistic. The c-statistic could be considered as the percentage of all possible pairs of cases in which the model assigns a higher probability to a correct case than to an incorrect case.

To assess the distribution of cases inside each *municipality*, the number of cases is assumed to have a Poisson distribution [9]: O_i _~Poisson (*μ*_*i*_). Therefore, the model is represented by the following equation:(1)

where log (*E*_*i*_) is treated as an offset, *α *is a constant, *x*_*i *_is an explanatory variable with coefficient *β *and *u*_*i *_represent the effects of the heterogeneity among the *municipalities*.

**Figure 1:**
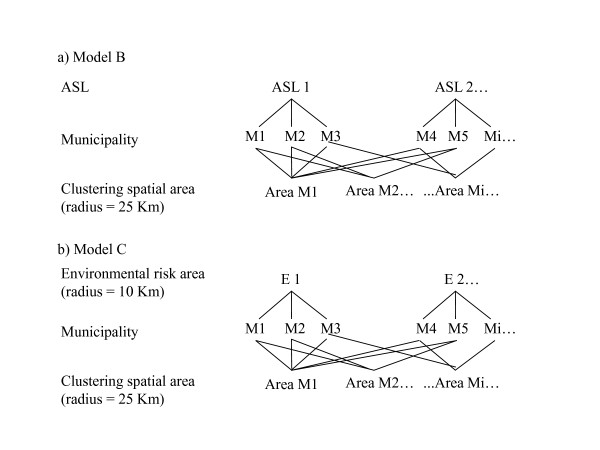
**Classification Diagrams of the"Multiple Membership Model"**.

In order to take into account the fact that relative risks can be spatially autocorrelated, the multilevel model must be seen as a "Multiple Membership Model" [10,11], where each *municipality *belongs to a higher level unit that also contains the neighboring *municipalities *(figure 1). The criterion used to establish the geographically neighboring units or cluster level could be adjacency or, as in our case, the choice of a distance radius (in km) within which all the *municipalities *are considered to belong to the same cluster. The first model to estimate (Model A) is a Multiple Membership Model:(2)

where *v*_*i *_represents the random effects due to the spatial dependency and *x*_*i*_*β *= 0 if there is no covariate.

Each *municipality i *is spatially dependent on one or more *municipalities j *belonging to the higher level geographic area ∂_i_, each of which contributes with weight z_ij_. The sum of the weights of *municipality i *is equal to one. Therefore, when drawing up the model each spatial effect *v*_i _referred to *municipality i *must be taken as the sum of a set of independent random effects, so that:(3)

 can be seen as the effect of *municipality j *on the other *municipalities *and z_ij _is its associated weight. If n_j _is the number of *municipalities *inside the geographic area ∂_*i *_(inside the cluster of *municipalities *with *municipality i *at its center), then: z_ij _= 1/n_j _if j∈∂_i _(*j *is one of the *municipalities *belonging to area *i*) z_*ij *_= 0 otherwise

Therefore, (2) yields the estimate in Model A, that becomes:(4)

If a suitable specification of the clustering and heterogeneity elements can be achieved, this has the effect of stabilizing the prevalence values and thus providing, for each *municipality*, estimated prevalence values that are a reasonable compromise between the observed value and a reference value. For the heterogeneity element the reference value is the mean prevalence in the general population (regional in this case), while for the clustering element this value is the mean prevalence in the neighboring *municipalities*.

In our first hypothesis the hospitalization rate varies among *municipalities *also according to the different management of the diagnosis by the local health service units. For this reason, we have added a further random effect *w*_*i *_representing the ASL each *municipality *belongs to:(5)

Given that **Z **= {*z*_*ij*_}, (3) can be written in matricial form:(6)

In multilevel models, the clustering variance estimated with (6) is obtained by means of linear combinations of single parameters of variance and can therefore take on negative values [12]. Even if this is mathematically possible, variance can never be negative and so the model needs to be re-estimated setting the clustering variance value at zero [13]. However, by doing so, when determining the smoothed rates no account is taken of the term representing the spatial contribution of the neighboring areas. The alternative is to estimate a model in which covariance *σ*_vu _is set at zero [14]. If the covariance terms between the random effects representing the ASL and the random effects of heterogeneity and spatial dependence are considered null, then the effects , *u*_*i *_e *w*_*i *_are assumed to be distributed according to the Normal Multivariates:(7)

Given (6) and considering that [15], (7) can be rewritten as follows:(8)

where **I **represents the identity matrix.

After building the matrix of random effects (8), Model B was estimated by (5) and the smoothed hospitalization rates for each *municipality *were calculated.

In the second hypothesis the hospitalization rates vary among *municipalities *according to the degree of exposure to some risk factors. We therefore identified 12 mutually esclusive areas of environmental risk, each centered around the *municipality *where industries with a high environmental impact are located, and extending for a radius of 10 km around it [16,17]. Then Model C was estimated by (5), with the random effect *w*_*i *_that represents the risk area in which the *municipality *is located. We compared the models using the Akaike Information Criterion (AIC). It is defined as 2 k - 2 ln(L), where k is the number of parameters in the model and ln(L) is the maximized log-likelihood of the model. The index takes into account both the statistical goodness of fit and the number of parameters that have to be estimated to achieve this particular degree of fit, by imposing a penalty for increasing the number of parameters. Lower values of the index indicate the preferred model, in other words the one with the fewest parameters that still provides an adequate fit to the data [18].

On the proposed maps, the 12 centers are indicated where industrial poles with a high environmental impact are located, to graphically illustrate their effect on the geographic distribution of the disease.

The total variance for a municipality depends on the number of neighboring municipalities and is expressed by  in Model A and  in Models B and C [15], where *n*_*i *_is the mean number of municipalities in the spatial dependency areas and *m*_*i *_the mean number of municipalities belonging to the ASL or the environmental risk areas in Models B and C, respectively. The term  expresses the spatially structured variability quota.

The links between the first and second levels of the hierarchical model can be summarized in a Classification Diagram reported in figure 1[19]. The 1^st ^level units are the *municipalities*, the 2^nd ^are on one hand the *municipalities *located within a radius of 25 km of the 1^st ^level unit, and on the other the ASL in Model B (Figure 1a) and the environmental risk areas in Model C (Figure 1b).

Results are considered statistically significant at a p-value < 0.05. Statistical analysis and mapping were performed using the packages BASE, STAT and GRAPH of SAS software Version 9.2 for PC.

### Data Used

The analysis was conducted using the HDF for Apulian residents for the year 2006 [7]. For each of the diseases studied, those patients admitted with a primary diagnosis of one of the ICD9-CM codes reported in Table 1 were selected.

**Table 1:** Selected ICD9-CM diagnosis codes for each disease, represented by spatial smoothing

Diagnosis code	Description
162 - -	Malignant tumors of the trachea, bronchi and lungs
49120 49121	Chronic Obstructive Bronchopneumonia

The rates were determined on the population recorded for the Apulia Region on the date 01/01/2007. The rates were calculated at the *municipal *level and to calculate the expected cases, a logistic model was used in the procedure of Risk Adjustment for age and gender. The distances among *municipalities *were calculated on the euclidean distance between the centroids of the areas of each *municipality *present on the map. The industrial centers posing an environmental risk were individuated on the basis of the INES register (national register of emission and their sources) for the year 2006, that refers information about emissions and sources of air pollutants such as COx, NOx, and PM (particulate matter), Pb, CrO etc..., from the steel industry, petroleum industry, thermoelectric industry.

Finally, the maps were shaded using the classification method of the quintiles of distribution of the rates [20].

## Results

### Lung Cancer

In 2006, a total of 2,591 patients resident in Apulia were hospitalized with a primary ICD9-CM diagnosis in the category "Malignant tumors of the trachea, bronchi and lungs", on a total resident population of 4,071,518 (crude regional rate = 6.36 per 10,000 inhabitants).

The logistic model to determine RAR resulted statistically significant (Wald test = 4232.7552; p < 0.0001) and c-statistic was 0.839, suggesting a good fit of the model.

The parameters and estimated standard errors with Models A, B and C are shown in Table 2.

**Table 2:** Parameters and estimated standard errors in the rates smoothing models

	Lung Cancer					
	**A - Model with spatial effect**		**B - Model with spatial effect and ASL effect**		**C - Model with spatial effect and risk Area effect**	
			
	**Estimate**	**St. Error**	**Estimate**	**St. Error**	**Estimate**	**St. Error**

*Fixed part *Intercept	0.0169	0.0596	-0.0369	0.0575	0.0826	0.0759
						
*Random part*						
*σ*^2^_u heterogeneity_	0.0336*	0.0144	0.0402*	0.0154	0.0272	0.0147
*σ*^2^_v clustering_	0.6940*	0.3025	0.2635	0.2498	0.7179*	0.3043
*σ*^2^_w ASL_			0.0218	0.0203		
*σ*^2^_w environmental risk area_					0.0064	0.0085
*n*_*i*_	*29.07*		*29.07*		*29.07*	
*m*_*i*_			*43.00*		*19.85*	
*σ*^2 ^_v_/*n*_*i*_	0.0239		0.0091		0.0247	
*σ*^2 ^_w_/*m*_*i*_			0.0005		0.0003	
*σ*^2 ^_TOTALE_	0.0575		0.0498		0.0522	
AIC	390.6		389.4		393.2	


	**COPD**					

	**A - Model with spatial effect**		**B - Model with spatial effect and ASL effect**		**C - Model with spatial effect and risk Area effect**	
			
	**Estimate**	**St. Error**	**Estimate**	**St. Error**	**Estimate**	**St. Error**

*Fixed part *Intercept	-0.1137	0.0953	-0.0327	0.1286	-0.1137	0.0953
*Random part*						
*σ*^2^_u heterogeneity_	0.0722*	0.0152	0.0755*	0.0147	0.0722*	0.0152
*σ*^2^_v clustering_	2.1485*	0.9170	0.7946	0.5500	2.1485*	0.9170
*σ*^2^_w ASL_			0.0747	0.0557		
*σ*^2^_w environmental risk area_					0.0000	-
*n*_*i*_	*29.07*		*29.07*		*29.07*	
*m*_*i*_			*43.00*		*19.85*	
*σ*^2^_v_/*n*_*i*_	0.0739		0.0273		0.0739	
*σ*^2^_w_/*m*_*i*_			0.0017		0.0000	
*σ*^2^_TOTALE_	0.1461		0.1045		0.1461	
AIC	311.4		297.4		311.4	

* p < 0.05

In Model A, both the variance due to the heterogeneity of the municipalities (*σ*^2^_u _= 0.0336, p = 0.0192) and clustering variance (*σ*^2^_v _= 0.6940, p = 0.0218) were statistically significant. Spatially structured variability in model A, obtained as the clustering variability estimate, divided by the mean number of municipalities in the spatial dependency areas *n*_*i *_= 29.07, resulted 0.0239. This result, summed with the heterogeneity variability, gives the total random variability for a municipality, 0.0575. Thus, the result of the spatially structured variability quota in model A was 41.57% (0.0239/0.0575).

In Model B, the random effect due to the ASL was added, whose estimated value was not significant (*σ*^2^_w ASL_= 0.0218, p = 0.1406); also the clustering variance resulted not statistically significant (*σ*^2^_v _= 0.2635, p = 0.2914). The only significant parameter was the variance due to the municipality heterogeneity (*σ*^2 ^_u _= 0.0402, p = 0.0092). The ASL variance for a municipality in model B, obtained as the ASL variance estimate, divided by the mean number of municipalities belonging to the ASL *m*_*i *_= 43.00, resulted 0.0005. This result, summed with the heterogeneity variance, and the clustering variance, 0.0091 (0.2635/29.07), gives the total random variability for a municipality, 0.0498. Thus, the spatially structured variability quota is lower: 18.27% (0.0091/0.0498), while the ASL value is equal to 1.00% (0.0005/0.0498). The AIC is slightly lower than in Model A.

In Model C the estimated random effect due to the environmental risk areas is not significant (*σ*^2^_w envir.risk.area _= 0.0064, p = 0.2261), nor is the heterogeneity variance, (*σ*^2^_u _= 0.0272, p = 0.0654), while the only significant parameter is the clustering variance (*σ*^2^_v _= 0.7179, p = 0.0183). The environmental risk area variability for a municipality in model C, obtained as the environmental risk area variance estimate, divided by the mean number of municipalities belonging to the environmental risk areas *m*_*i *_= 19.85, resulted 0.0003. This result, summed with the heterogeneity variance and clustering variance, 0.0247(0.7179/29.07), gives the total random variance for a municipality, 0.0522. Thus, the spatially structured variability quota is equal to 47.32% (0.0247/0.0522) and the environmental risk area variability is 0.57% (0.0003/0.0522). The AIC is higher than in Model A.

**Figure 2:**
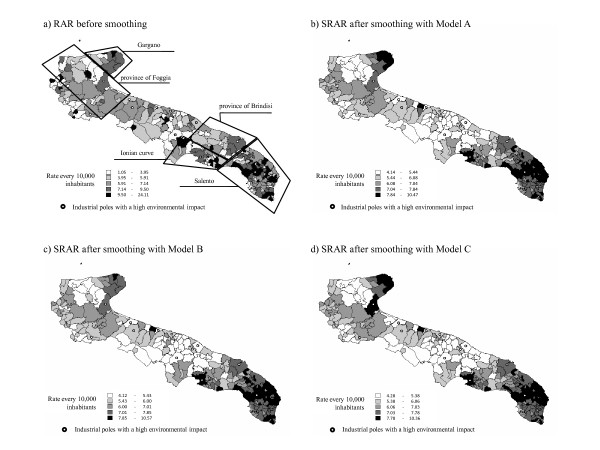
**Maps of the Hospitalization Rate for Lung cancer**. Apulia (Italy), 2006.

Four maps were built: the first one using the rates obtained at the end of the Risk Adjustment procedure before smoothing and the second, third and fourth using the smoothed rates obtained after estimating Models A, B and C, respectively (figure 2). The map in figure 2a does not offer a clear visual picture of areas with higher or lower hospitalization rates for lung cancer.

In figure 2b it can be seen that there is a tendency to clustering of municipalities with a higher admission rate for lung cancer in the Salento, the southernmost part of the Ionian curve and the Gargano, whereas the zones in the central part of the region (with the exception of some coastline municipalities) show low rates of hospitalization; this applies to the northern part of the province of Foggia, too.

In figure 2c the introduction of the random effect of the ASL changes the level of hospitalization rates in several municipalities as compared to figure 2b. In particular, a reduction of the hospitalization rates for lung cancer on the Gargano seems to appear.

On the contrary, in figure 2d the introduction of the random effect of the areas at environmental risk produces little variation in the appearance of the municipalities hospitalization rate level as compared to figure 2b. The Gargano area is differently evidenced in figure 2c and figure 2d, where the latter gives the appearance of high rates for this area, probably due to the effect of environmental factors included in Model C and because few municipalities with higher rates are aggregated, as compared to the municipalities aggregated in Model B.

We have indicated the industrial centers posing an environmental risk on the maps. In the maps with smoothed rates (Figures 2b, 2c, 2d), the areas with the higher admission rates are centered around municipalities with large industrial plants (such as Taranto in the Ionian curve) suggesting the presence of risk factors, both environmental and professional, that can explain higher rates of hospitalization for lung cancer.

**Figure 3:**
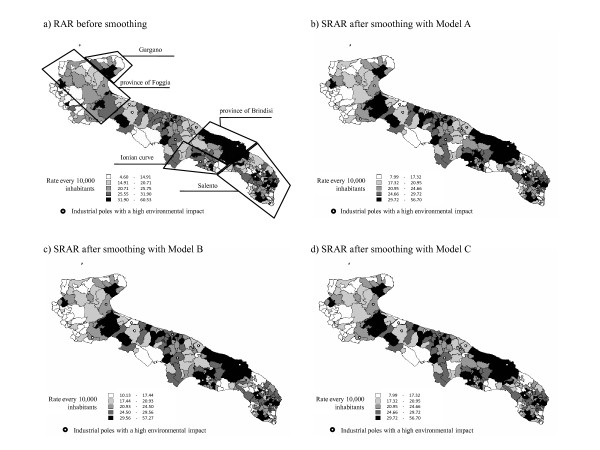
**Maps of the Hospitalization Rate for COPD**. Apulia (Italy), 2006.

Figure 3 shows the differences between the RAR and the smoothed RAR (SRAR) obtained with Model A, for the geographic surface of the municipalities expressed in square km (sqKm). The effect of smoothing for spatial dependence on the hospitalization rates is greater for smaller *municipalities *with a surface area of less than 100 sqKm (Figure 3a). The same graph, built calculating the differences between the RAR and SRAR in Model B and Model C, produces comparable results to those shown in Figure 3, so these data are not shown.

**Figure 4:**
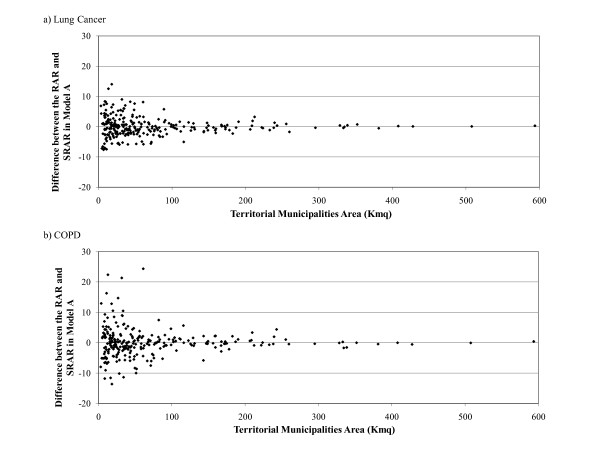
**Difference between the RAR and SRAR in Model A by Territorial Municipalities Area (sqKm)**.

In Figure 4 the differences between the RAR and SRAR obtained with Model A are compared with the population at risk. The smaller the number of people at risk, the greater the difference between the RAR and SRAR (Figure 4a).

### Chronic Obstructive Pulmonary Disease

In 2006, 10,356 patients resident in Apulia were hospitalized with a primary diagnosis of one of the two ICD9-CM diagnostic codes for "Chronic Obstructive Pulmonary Disease", on a total resident population of 4,071,518 (crude regional rate = 25.43 per 10,000 inhabitants).

The logistic model to determine the RAR resulted statistically significant (Wald test = 14225.7969; p < 0.0001) and the c-statistic was 0.872, suggesting a good fit of the model.

In Model A both elements of variance were significant (table 2): heterogeneity variance (*σ*^2^_u _= 0.0722, p < 0.0001) and clustering variance (*σ*^2^_v _= 2.1485 p = 0.0191).

Spatially structured variability in model A, obtained as the clustering variance estimate, divided by the mean number of municipalities in the spatial dependency areas *n*_*i *_= 29.07, resulted 0.0739. This result, summed with the heterogeneity variance, gives the total random variability for a municipality, 0.1461. Thus, the clustering variance accounts for 50.58% (0.0739/0.01461) of the total variance.

In Model B, only the heterogeneity variance is significant (*σ*^2^_u _= 0.0755, p < 0.0001). The ASL variance estimate (*σ*^2^_ASL _= 0.0747, p = 0.0902) and clustering variance (*σ*^2^_v _= 0.7946, p = 0.1485) did not result statistically significant. The ASL variability for a municipality in Model B, obtained as the ASL variability estimate, divided by the mean number of municipalities belonging to the ASL *m*_*i *_= 43.00, resulted 0.0017. This result, summed with the heterogeneity variance, and clustering variance, 0.0273 (0.7946/29.07), gives the total random variance for a municipality, 0.1045. Thus, the spatially structured variability quota is 26.12% (0.0273/0.1045), notably smaller than in model A, while the ASL quota is 1.63% (0.0017/0.1045). In Model B the AIC is much better than in Model A. In Model C the estimated variance for the environmental risk areas is equal to zero, so all the other parameters are the same as in Model A.

**Figure 5:**
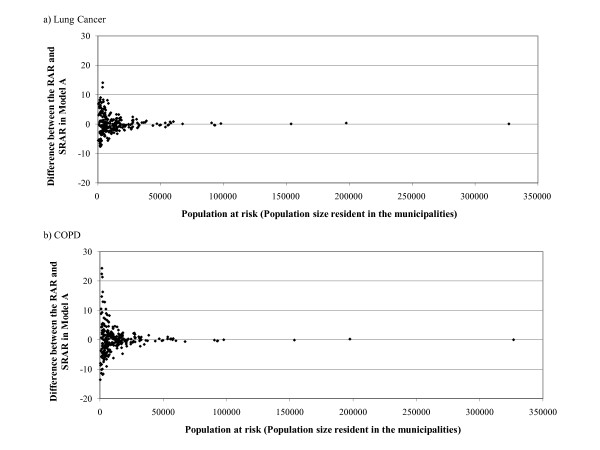
**Difference between the RAR and SRAR in Model A by Population size resident in the Municipalities**.

If we look at the map of the SRAR obtained with Model A (Figure 5b), we can see a higher hospitalization rate for COPD in the province of Brindisi, confirming what was shown by the map built before smoothing (Figure 5a). Introduction of the hierarchical level of the ASL (Figure 5c) barely affects the shading at *municipal *level as compared with that in Figure 5b, and is not relevant in terms of depicting a different distribution of higher and lower areas of risk for hospitalization. Since the introduction of the random effect of the environmental risk areas did not change the estimated parameters, the map in Figure 5d is identical to that in Figure 5b.

The smoothed maps show a higher admission rate around the large industrial plants in the Ionian curve and Brindisi province, but not differently from the RAR, or model A. Perhaps in the case of COPD the higher number of cases in the whole territory results less sensitive to smoothing.

The difference between the RAR and SRAR was very high in small *municipalities *(Figure 4b) and those with a small number of residents (Figure 4b).

## Discussion and Conclusion

The aim of this work was to evaluate the effect of smoothing for two chronic diseases, based on the assumption that hospitalization rates may be influenced by both the neighboring municipalities and the health service organization or environmental risk factors associated with the disease under study.

In such cases, a simple depiction of the rates adjusted by Risk Adjustment techniques might not be sufficiently representative.

From the graphic standpoint, the smaller the area on which the indicator is calculated, the better the spatial representation. However, in this case there is a greater risk of instability of the indicator, due to the small number of cases observed.

Furthermore, to estimate correlations of rates among municipalities we adopted multilevel model analytical techniques [21] that take into account spatial dependence among neighboring areas, subdividing the variability among municipalities into a structured component representing this spatial dependence, and another non structured component representing their heterogeneity [22-24]. In this way we obtained a smoothed value of the indicator that tended to provide a more meaningful representation of the true risk in each area, and especially in smaller areas.

To build the variance and covariance matrixes the weights used were determined considering the number of municipalities included in an assigned radius. Other formulations can also be used to determine the weights, such as those introducing a distance function, but in other experiences difficulties with the methods and results of estimation arose [25-27].

The choice of a radius of 25 km to identify a level of aggregation is justified by the fact that it is the median distance between neighboring municipalities. A more objective solution to the aggregation could be researched based on a function of rates distribution. The choice of a smaller distance than 25 km increases the number of aggregate areas with few municipalities, causing an increase of the units in the second hierarchical levels that could overlap first level units. A distance of more than 25 km determines large aggregations with the effect of lowering rates, if the aggregation includes a high number of municipalities with few cases.

Both for lung cancer and for COPD, the results of estimated models in which the clustering and heterogeneity components were adequately specified demonstrated that both heterogeneity and spatial autocorrelation were significant parameters. This is understandable because the municipalities are characterized by fairly variable demographic data. In fact, the effect of the smoothing procedure was greater in smaller municipalities and especially in those with a more unstable RAR value due to the small number of cases and of population at risk.

The addition of a level representing the areas at environmental risk among the random effects of the Spatial Multimembership Model, for lung cancer and COPD, did not have significant effects on the subdivision of the variability between the structured and the heterogeneity components. Instead, when the local health service organization (ASL) was considered as a second hierarchical level parallel to that of spatial dependency, the municipalities heterogeneity component increased markedly for both diseases and the model fitted the data better, especially as regards COPD.

In view of the territorial variability of the risks (estimated by the SRAR), the map of hospitalization rates for lung cancer in the Apulian Region revealed the areas at higher risk, unlike the maps estimated by the RAR, where the visual impact was less immediate. The number of cases, and hence the hospitalization rate, is greater for COPD than for lung cancer, giving rise to more stable municipalities rates, so the graphic effect of the smoothing procedure for this disease was less evident. From the graphic standpoint, the insertion of areas at environmental risk did not significantly change the degrees of shading of the map and hence the depiction of the risk. On the contrary, the inclusion of the ASL changed the spatial distribution of the risks, especially for lung cancer, demonstrating a reduced hospitalization rate in the Gargano zone. This is probably due to the different organization in this ASL, where there is a lower propensity to admit patients to hospital and a lower availability or accessibility of diagnostic services, as compared with other ASL.

Inclusion in the model of a hierarchical level representing the industrial areas with a strong environmental impact was found to be redundant, even for those diseases already proven to be correlated to pollutant atmospheric agents. In fact, capturing the spatial autocorrelation was enough to depict a concentration of the areas at higher risk precisely in those areas centered around industries producing the emission of harmful substances.

These results show that unlike the ASL, the environmental risk is not a better hierarchical level than the "municipality" but rather an attribute of the municipality itself, representing the risk factor posed by its vicinity to highly polluting industrial plants. The contribution of the environmental risk is probably better studied by inserting it in the model as a covariate. Moreover, other more specific information on the degree of exposure to environmental risk factors, like the type and quantity of airborne polluting substances and fine particles released by the industrial centers present in the area under study, as well as meteorological factors, could explain higher quotas of residual variability and provide another useful contribution to the graphic representation of the hospitalization rates.

To gain the best interpretation of the specific results for the diseases analyzed, it must be borne in mind that some of the remarkable results might appear different when taking into account the estimated risks for the neighboring Italian regions. We can aggregate neighboring municipalities to smooth boundary rates. Furthermore, the availability of standard national rates could be useful to compare our results for this region and reweight observed rates in the right setting.

As concluded by Zhou et al., 2008 [28], after building smoothed risk maps, it would be useful to explore the potential reasons for the clustering observed, such as the socio-economic factors and the medical practice present and characterizing the areas under study.

## Competing interests

The authors declare that they have no competing interests.

The research is partially funded with Prin-University 2007 grant.

## Authors' contributions

NB conceived the study, conducted the analysis, wrote the manuscript. PT conceived the study, collaborated in the analysis, drafted the manuscript. GS supervised the analysis and reviewed the manuscript. All authors read and approved the final manuscript.
